# Five Years Later: An Update on the Status of Collections of Endemic Gulf of Mexico Fishes Put at Risk by the 2010 Oil Spill

**DOI:** 10.3897/BDJ.4.e8728

**Published:** 2016-08-18

**Authors:** Prosanta Chakrabarty, Glynn A. O’Neill, Brannon Hardy, Brandon Ballengee

**Affiliations:** ‡ Louisiana State Unviersity Museum of Natural Science, Baton Rouge, Louisiana, United States of America; § National Science Foundation, Arlington, Virginia, United States of America; | Louisiana State University, Baton Rouge, United States of America; ¶ Louisiana State Unviersity Museum of Natural Science, Baton Rouge, United States of America

**Keywords:** BP, Deepwater Horizon, Macondo, ichthyology, fish

## Abstract

**Background:**

The 2010 Gulf of Mexico Oil Spill took place over 180,000 square kilometers during a 12-week period over five years ago; however, this event continues to influence the development and distribution of organisms in and around the region of the disaster. Here we examine fish species that may have been most affected by noting their past distribution in the region of the spill and examining data of known collecting events over the last 10 years (five years prior to the spill, five years post spill).

**New information:**

We found that more than half of the endemic fish species of the Gulf (45 of 77)

## Introduction

The 2010 Gulf of Mexico Oil Spill (also called the Deepwater Horizon/BP disaster/oil spill, or Macondo blowout among others) was the largest accidental spill of oil in history (Crone and Tolstoy 2010, Rabalais 2014). Coupled with the fact that it occurred in the deep sea (>1000 m depth) and with the coordinated release of more than a million gallons of dispersant, it is one of the greatest pollution events in history (Goodbody-Gringley et al. 2013). The long lasting effects of the spill are still not fully understood even five years after the event. There is considerable evidence that some species continue to be physically and developmentally challenged by the impact of the spill, particularly fishes (Whitehead et al. 2011; Incardona et al. 2014; Dubansky et al. 2013; Brette et al. 2014; Mager et al. 2014; Alloy et al. 2016). However, population studies of fishes remain poorly examined (Fodrie et al. 2014). Although, fisheries for commercial species are better studied, the ichthyofauna as a whole has received little attention. Chakrabarty et al. (2012) listed fish species in need of conservation concern based on their known distribution in relation to the historical surface position of the oil spill. Here we reexamine the distribution of all 77 known endemic Gulf fish species five years after the spill based on collection records (as a reminder endemic means in this context, species only found in the Gulf of Mexico). We compare these post-spill records with those from five years prior to the spill.

These collection records are obtained from natural history museum records of specimen collections. Museum collections are a vital source for biological records (Drew 2011; Rocha et al. 2014). They maintain a record of the world’s biodiversity by keeping specimens recorded from a certain area and time allowing comparisons to be made across time and space. With these collections one can compare a changing fauna before and after a catastrophic event, such as an oil spill. The correct identification of specimens is also vital (Chakrabarty et al. 2013), as museum collections are maintained by taxonomists and the specimens and comparative material are at hand, the identifications from these collections are more trustworthy than those from ship records or other sources where specimens are discarded. Here we use these collection records to examine the affects of the 2010 Gulf of Mexico Oil Spill on the endemic fishes of the region.

## Methods and Results

The occurrence records of the 77 endemic species of the Gulf of Mexico were tallied using The Global Biodiversity Information Facility and FishNet2 from October-December of 2015. Duplicate events from the two databases were deleted (duplicates were discovered if they had the same museum catalog numbers). A scatter plot graph was then created in Microsoft Excel showing collections five years prior to the 2010 Oil Spill and five years post spill. Only collections records from the Gulf of Mexico were counted (assuming for these endemics that records from outside the region are likely misidentifications).

Scatter plots of endemic fishes from the Gulf of Mexico are shown below with the “Number of Occurrence(s)” on the y-axis vs. the “Number of Years” on the x-axis. Species are listed in alphabetical order. Endemic species that have few or no collections records do not have a scatterplot but details about their last collecting events are presented. The scientific name is also presented followed by common name (when there is one) and family. Spill zone overlap information is from Chakrabarty et al. (2012). If the scientific name has changed in the past five years we show both the old and new names. Conservation information about “Resilience” is taken from FishBase (Froese and Pauly 2016). Resilience is based upon the time it takes to double the species population and are as follows: Very Low (minimum of 14 years to double population); Low (4.5-14 years to double population); Medium (1.4-4.4 years to double population); High (less than 15 months to double population).

**Figure 1.**
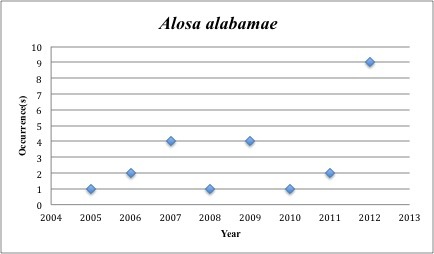
*Alosa alabamae*

**Figure 2.**
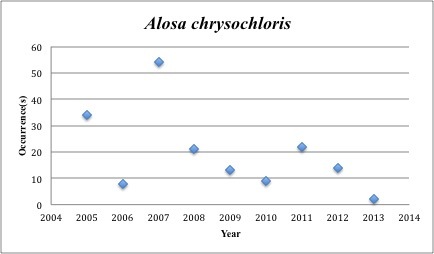
*Alosa chrysochloris*

**Figure 3.**
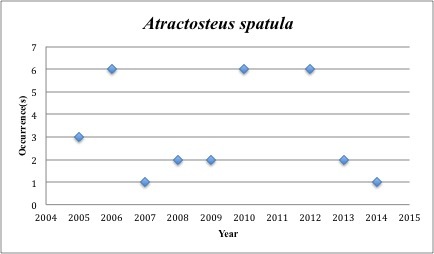
*Atractosteus spatula*

**Figure 4.**
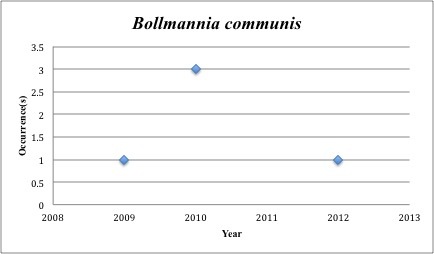
*Bollmannia communis*

**Figure 5.**
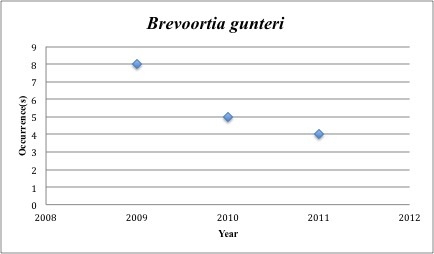
*Brevoortia gunteri*

**Figure 6.**
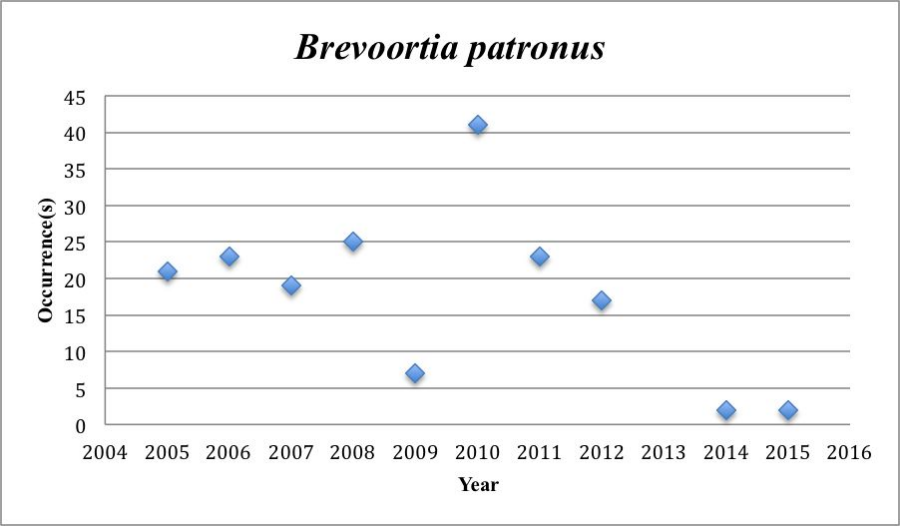
*Brevoortia patronus*

**Figure 7.**
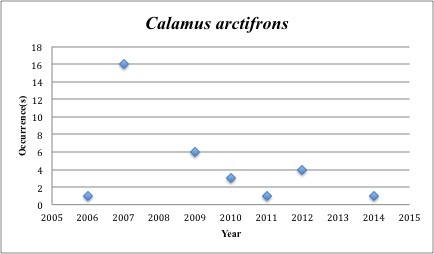
*Calamus arctifrons*

1) *Alosa alabamae* - Alabama Shad – Clupeidae (1% range overlap with spill zone). Resilience: Medium (Fig. 1)

2) *Alosa chrysochloris* - Skipjack Shad – Clupeidae (2% range overlap with spill zone). Resilience: Medium (Fig. 2)

3) *Anacanthobatis folirostris* - Leaf-nose Leg Skate – Anacanthobatidae (79% range overlap with spill zone). Resilience: Low. – last time collected: 2004

4) *Atherinella schultzi* - Chimalapa Silverside – Atherinopsidae (No range overlap with spill zone). Resilience: High – collected once (2013) since 2005

5) *Atractosteus spatula* – Alligator Gar – Lepisosteidae (No range overlap with spill zone). Resilience: Very low (Fig. 3)

6) *Bollmannia communis* – Ragged Goby – Gobiidae (41% range overlap with spill zone). Resilience: High (Fig. 4)

7) *Bollmannia eigenmanni* – Shelf Goby – Gobiidae (64% range overlap with spill zone). Resilience: Medium – last time collected: 1988

8) *Brevoortia gunteri* – Finescale Menhaden – Clupeidae (2% range overlap with spill zone). Resilience: Medium (Fig. 5)

9) *Brevoortia patronus* – Gulf Menhaden – Clupeidae (11% range overlap with spill zone). Resilience: Medium (Fig. 6)

10) *Calamus arctifrons* – Grass Porgy – Sparidae (No range overlap with spill zone). Resilience: Medium (Fig. 7)

11) *Calamus campechanus* – Campeche Porgy – Sparidae (No range overlap with spill zone). Resilience: Medium – last time collected: 1987

12) *Chasmodes longimaxilla* – Stretchjaw Blenny – Blenniidae (No range overlap with spill zone). Resilience: High – last time collected: 1983

13) *Chriolepis benthonis* – Deepwater Goby – Gobiidae (No range overlap with spill zone). Resilience: High – last time collected: 1953

14) *Chriolepis vespa* – Wasp Goby – Gobiidae (No range overlap with spill zone). Resilience: High – last time collected: 1970

15) *Citharichthys abbotti* – Veracruz Whiff – Paralichthyidae (No range overlap with spill zone). Resilience: High – last time collected: 2001

16) *Coryphaenoides mexicanus* – Mexican Grenadier – Macrouridae (54% range overlap with spill zone). Resilience: Medium (Fig. 8)

17) *Coryphopterus punctipectophorus* – Spotted Goby – Gobiidae (No range overlap with spill zone). Resilience: High – last time collected: 1982

18) *Ctenogobius claytonii* – Mexican Goby – Gobiidae (No range overlap with spill zone). Resilience: High – collected once (2005) since 2005

19) *Cynoscion arenarius* – Sand Weakfish – Sciaenidae (12% range overlap with spill zone). Resilience: Medium (Fig. 9)

20) *Dipturus olseni* – Spreadfin Skate – Rajidae (29% range overlap with spill zone). Resilience: Low – collected twice (2005) since 2005

21) *Dipturus oregoni* – Hooktail Skate – Rajidae (80% range overlap with spill zone). Resilience: Low – last time collected: 1987

22) *Eptatretus minor* – Hagfish – Myxinidae (23% range overlap with spill zone). Resilience: Low – collected twice (2005) since 2005

23) *Eptatretus springeri* – Gulf hagfish – Myxinidae (54% range overlap with spill zone). Resilience: Low – collected once (2010) since 2005

24) *Etmopterus schultzi* – Fringefin Lanternshark – Etmopteridae (90% range overlap with spill zone). Resilience: Low – collected five times (2006) since 2005

25) *Eustomias leptobolus* – Stomiidae (40% range overlap with spill zone). Resilience: High – last time collected: 1960

26) *Exechodontes daidaleus* – Zoarcidae (No range overlap with spill zone). Resilience: High – last time collected: 1989

27) *Floridichthys carpio* – Goldspotted killifish – Cyprinodontidae (No range overlap with spill zone). Resilience: High (Fig. 10)

28) *Fundulus grandis* – Gulf Killifish – Fundulidae (13% range overlap with spill zone). Resilience: High (Fig. 11)

29) *Fundulus jenkinsi* – Saltmarsh Topminnow – Fundulidae (4% range overlap with spill zone). Resilience: High (Fig. 12)

30) *Fundulus persimilis* – Yucatán Killifish – Fundulidae (No range overlap with spill zone). Resilience: High – collected twice in 2005

31) *Fundulus pulvereus* – Bayou Killifish – Fundulidae (18% range overlap with spill zone). Resilience: High (Fig. 13)

32) *Fundulus xenicus* (formerly *Adinia xenica*) – Diamond Killifish – Fundulidae (13% range overlap with spill zone). Resilience: Low (Fig. 14)

33) *Gambusia yucatana* – Yucatan Mosquitofish – Poeciliidae (No range overlap with spill zone). Resilience: High (Fig. 15)

34) *Gobiosoma longipala* – Twoscale Goby – Gobiidae (No range overlap with spill zone). Resilience: High – collected 2 times (2012) since 2005

35) *Gordiichthys ergodes* – Irksone Eel – Ophichthidae (No range overlap with spill zone). Resilience: Medium (Fig. 16)

36) *Gordiichthys leibyi* – String Eel – Ophichthidae (No range overlap with spill zone). Resilience: Medium – last time collected: 2004

37) *Gunterichthys longipenis* – Gold Brotula – Bythitidae (88% range overlap with spill zone). Resilience: Low – last time collected: 2002

38) *Gymnachirus texae* – Gulf of Mexico Fringed Sole – Achiridae (16% range overlap with spill zone). Resilience: High – collected once (2012) since 2005

39) *Halichoeres burekae* – Mardi Gras Wrasse – Labridae (No range overlap with spill zone). Resilience: High – collected twice (2006) since 2005

40) *Halieutichthys intermedius* – Louisiana Pancake Batfish – Ogcocephalidae (68% range overlap with spill zone). Resilience: High – collected five times (2010) since 2005

41) *Heteroconger luteolus* – Yellow Garden Eel – Congridae (No range overlap with spill zone). Resilience: Medium – last time collected: 2004

42) *Hyperoglyphe bythites* – Black Driftfish – Centrolophidae (82% range overlap with spill zone). Resilience: Medium – collected once (2008) since 2005

43) *Hypleurochilus caudovittatus* – Zebratail Blenny – Blenniidae (Insufficient data) Resilience: High – last time collected: 2004

44) *Hypleurochilus multifilis* – Featherduster Blenny – Blenniidae (25% range overlap with spill zone). Resilience: High – last time collected: 2001

45) *Ijimaia antillarum* – Ateleopodidae (8% range overlap with spill zone). Resilience: Unknown – last time collected: 2004

46) *Jordanella floridae* – Flagfish – Cyprinodontidae (No range overlap with spill zone). Resilience: Low (Fig. 17)

47) *Jordanella pulchra* (previously *Garmanella pulchra*) – Yucatán flagfish – Cyprinodontidae (No range overlap with spill zone). Resilience: High – collected 10 times (2005) since 2005

48) *Lepisosteus oculatus* – Spotted Gar – Lepisosteidae (0.2% range overlap with spill zone). Resilience: Medium (Fig. 18)

49) *Leucoraja lentiginosa* – Freckled Skate – Rajidae (53% range overlap with spill zone). Resilience: Low – collected once (2012) since 2005

50) *Lupinoblennius nicholsi* – Highfin Blenny – Blenniidae (No range overlap with spill zone). Resilience: High – last time collected: 2000

51) *Lycenchelys bullisi* – Zoarcidae (50% range overlap with spill zone). Resilience: Medium – last time collected: 1999

52) *Menidia clarkhubbsi* – Texas Silverside – Atherinopsidae (No range overlap with spill zone). Resilience: High – last time collected: 2000

53) *Menidia colei* –Golden Silverside – Atherinopsidae (No range overlap with spill zone). Resilience: High – collected 29 times (2005) since 2005

54) *Menidia conchorum* – Key Silverside – Atherinopsidae (No range overlap with spill zone). Resilience: High – last time collected: 1978

55) *Microdesmus lanceolatus* – Lancetail Wormfish – Microdesmidae (43% range overlap with spill zone). Resilience: High – last time collected: 1980

56) *Monopenchelys acuta* – Redface Moray – Muraenidae (No range overlap with spill zone). Resilience: High (Fig. 19)

57) *Mustelus sinusmexicanus* – Gulf Smooth-hound – Triakidae (43% range overlap with spill zone). Resilience: Low (Fig. 20)

58) *Neoopisthopterus cubanus* – Cuban Longfin Herring – Pristigasteridae (Insufficient data). Resilience: High – last time collected: N/A

59) *Ogcocephalus pantostictus* – Spotted Batfish –Ogcocephalidae (3% range overlap with spill zone). Resilience: Low (Fig. 21)

60) *Ogilbia cayorum* – Key Brotula – Bythitidae (No range overlap with spill zone). Resilience: Low (Fig. 22)

61) *Oneirodes bradburyae* – Oneirodidae (100% range overlap with spill zone). Resilience: High – last time collected: 1954

62) *Ophichthus omorgmus* – Dotted Snake Eel – Ophichthidae (Insufficient data). Resilience: Medium – last time collected: 1999

63) *Ophichthus rex* – King Snake Eel – Ophichthidae (82% range overlap with spill zone). Resilience: Very low – collected once (2009) since 2005

64) *Opsanus pardus* – Leopard Toadfish – Batrachoididae (38% range overlap with spill zone). Resilience: Low (Fig. 23)

65) *Parasaccogaster rhamphidognatha* (previously *Saccogaster rhamphidognatha*) – (100% range overlap with spill zone). Resilience: High – last time collected: N/A

66) *Parmaturus campechiensis* – Campeche Catshark – Pentanchidae (Insufficient data). Resilience: Low – last time collected: 1970

67) *Prionotus longispinosus* – Bigeye Sea Robin – Triglidae (50% range overlap with spill zone). Resilience: Medium (Fig. 24)

68) *Prionotus martis* – Gulf of Mexico Barred Sea Robin – Triglidae (5% range overlap with spill zone). Resilience: High (Fig. 25)

69) *Prionotus paralatus* – Mexican Sea Robin – Triglidae (Insufficient data). Resilience: High (Fig. 26)

70) *Raja texana* – Roundel Skate – Rajidae (11% range overlap with spill zone). Resilience: Low (Fig. 27)

71) *Sanopus reticulates* – Reticulate toadfish – Batrachoididae (Insufficient data). Resilience: Medium – last time collected: 1977

72) *Sphoeroides parvus* – Least Puffer – Tetraodontidae (Insufficient data). Resilience: High (Fig. 28)

73) *Sphoeroides spengleri* – Bandtail Puffer – Tetraodontidae (.4% range overlap with spill zone). Resilience: High (Fig. 29)

74) *Stemonosudis bullisi* – Paralepididae (Insufficient data). Resilience: High – last time collected: 1960

75) *Syngnathus affinis* – Texas Pipefish – Syngnathidae (No range overlap with spill zone). Resilience: High – last time collected: 1983

76) *Trichopsetta ventralis* – Sash Flounder – Bothidae (31% range overlap with spill zone). Resilience: Medium (Fig. 30)

77) *Varicus marilynae* – Orangebelly Goby – Gobiidae (No range overlap with spill zone). Resilience: High – last time observed: 1974

## Discussion

The continued influence of an oil spill that occurred more than five years ago on the Gulf of Mexico is evident (Incardona et al. 2014; Alloy et al. 2016; Schaefer et al. 2015); however, data about population status, or even tangible proof of the continued existence of many of the Gulf’s endemic fish species, is lacking. More than half (45) of the 77 endemic species from the Gulf of Mexico have not been officially collected since the 2010 spill. Of these, nine species have not been collected since before 1980, eight species have not been collected since the 1980s, and two not since the 1990s. Although there is a focus on fisheries data for commercially important species post-spill, the endemic species examined here are among the Gulf species we know the least about. Even with the data presented here our study of collections records must be viewed as a small glimpse into the true effects of the spill. Collections records are not a true estimate of population dynamics; however, in the case of rare and poorly studied species (as is the case with these endemics) – it is our best estimate.

The species we should perhaps be most concerned for are the 14 that have collection records in the five years before the spill, but lack records post-spill (2010-2015). Among these are *Fundulus jenkinsi* ­collected 306x, *Menidia colei* (29x), *Jordanella pulchra* (10x), *Ogilbia cayorum* (6x), and *Etmopterus schultzi* and *Monopenchelys acuta* both collected 5x. *Gambusia yucatana* was collected 14x in the last 10 years, and all but one of those was pre-spill.

Other species appear to be more common post-spill, with most of the collections occuring in the last five years (rather than the 2005-2010 period): *Trichopsetta ventralis* (6 of 8 collections post-2010), *Sphoeroides parvus* (83 of 109), *Prionotus longispinous* (203 of 206), *Prionotus paralatus* (74 of 76), *Opsanus pardus* (6 of 7), *Ogcocephalus pantostictus* (6 of 6), *Gobiosoma longipala* (2 of 2). It should be noted that all the collections of *Halieutichthys intermedius* are post-spill because this species was described in 2012 (Ho et al. 2012) and most museums have not updated their records for this species. Some of the species that had higher collections numbers post spill may have been influenced by the closing of fisheries during and after the immediate period of the oil spill (Schaefer et al. 2015). Although not directly targeted for fisheries these species may have increased in number because they were not collected as by-catch when fishing was closed. Also the increased interest in collecting and studying Gulf species post spill may have increased efforts to identify and catalogue these species. We also note here that the collections efforts pre- and post-spill were likely not equal. We therefore cannot do a statistical sampling comparison based on collecting effort.

There are some notable trends among and within groups as well. Of the six eels in the study (Elopomorpha Families: Ophichthidae, Muraenidae, Congridae) only one species, *Ophichthus rex* had a high percentage of its range in the region of the spill (82%) and it has been collected once since the spill. However, eel species in general are very rare in collections, and little or no data about any of the endemic eels from the Gulf of Mexico is known (9 total collection records, all post spill).

Of the seven cartilaginous fishes (Elasmobranchii Families: Anacanthobatidae, Rajidae, Etmopteridae, Triakidae) most had a high proportion of their range in the area of the spill zone but most have post-spill collections. The exception being the rare *Anacanthobatis folirostris*, which has no collection records since 2004. These elasmobranchs all have low resiliency, with populations doubling time between 4.5-14 years (Froese and Pauly 2016). Most members of the small but diverse members of gobies (Gobioidei) and blennies (Blennioidei) lack sufficient information (in being collected mostly before 2005), as is the case for most of the ten coral associated endemic Gulf species (Table 1). Inshore brackish fishes such as those in the families Lepisosteidae, Clupeidae, Atherinopsidae, Fundulidae, Poeciliidae, and Cyprinodontidae, were mainly out of the area of the immediate spill (i.e., little overlap with the region of the spill as initially measured) and are among the most collected species among Gulf endemics (Table 1​). However, although the collections may be high, the documented developmental impairment of near shore species points to the fact that even these species are not out of harms way (Dubansky et al. 2013). Additionally, the influence of the oil slick at the surface on pelagic larvae and in the deep-sea on individuals that are rarely seen will never be completely known (Fodrie and Heck 2011).

More than quarter of the Gulf of Mexico endemic fish species (20) had greater than 35% of their historical records in the area of the spill zone (Chakrabarty et al. 2012; those in bold text in Table 1). These species were identified by Chakrabarty et al. (2012) as being in the highest potential impact category. Of these species half (10 species) still lack any collection records post spill. We note that both GBIF and FishNET are not perfect records of all collecting events or even all museum collections. Also we note that these databases are dynamic and change on a near daily basis as museum records are uploaded and updated. For that reason the data in this paper should be taken as a snapshot of the information available at this time. It is clear more work needs to be done to find and potentially protect these endemic taxa. Future work will include citizen science projects by the authors (see Acknowledgements) and others, that will target Gulf endemics and add data, museum records, and increase community awareness. We hope this study helps focus conservation efforts on those species that lack the most information, or that have not been collected post-spill.

## Figures and Tables

**Figure 8.**
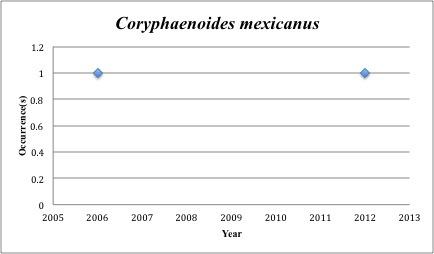
*Coryphaenoides mexicanus*

**Figure 9.**
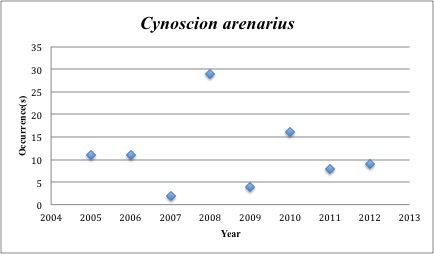
*Cynoscion arenarius*

**Figure 10.**
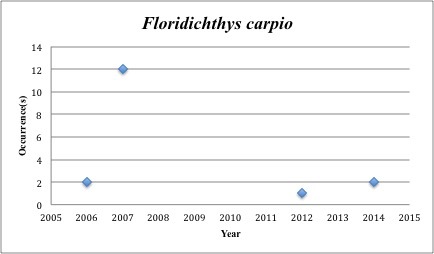
*Floridichthys carpio*

**Figure 11.**
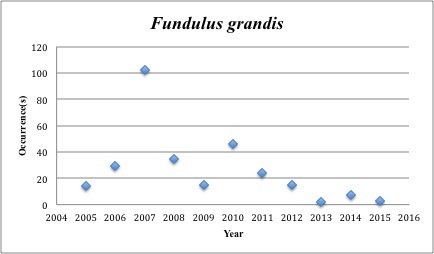
*Fundulus grandis*

**Figure 12.**
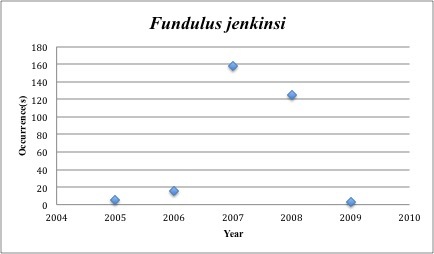
*Fundulus jenkinsi*

**Figure 13.**
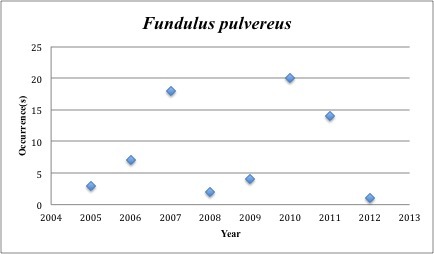
*Fundulus pulvereus*

**Figure 14.**
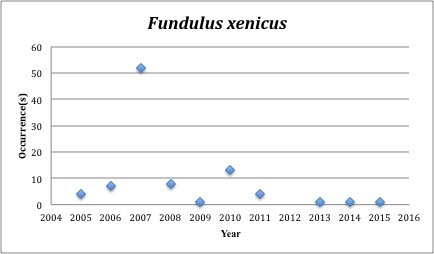
*Fundulus xenicus*

**Figure 15.**
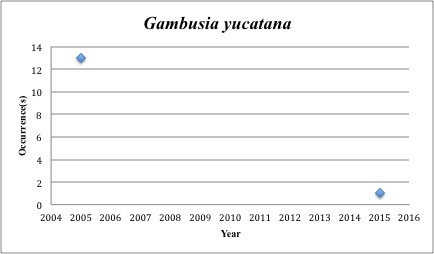
*Gambusia yucatana*

**Figure 16.**
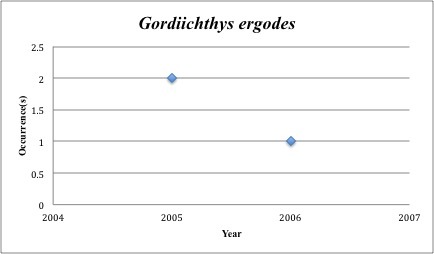
*Gordiichthys ergodes*

**Figure 17.**
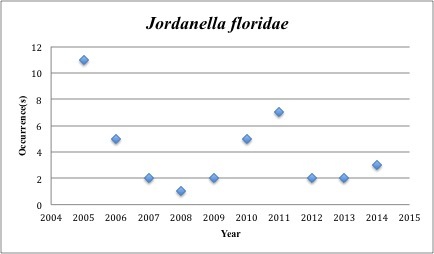
*Jordanella floridae*

**Figure 18.**
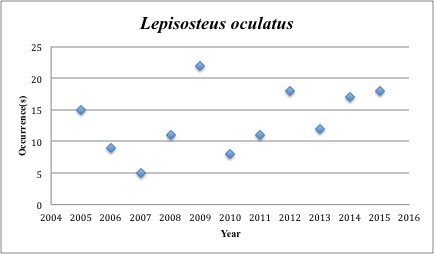
*Lepisosteus oculatus*

**Figure 19.**
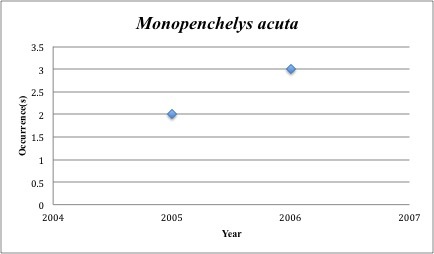
*Monopenchelys acuta*

**Figure 20.**
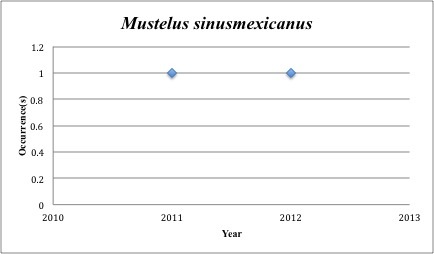
*Mustelus sinusmexicanus*

**Figure 21.**
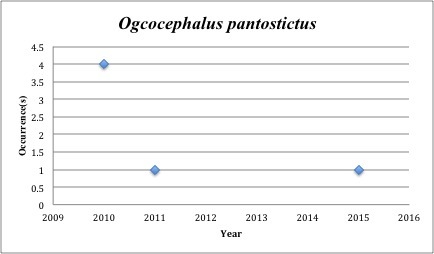
*Ogcocephalus pantostictus*

**Figure 22.**
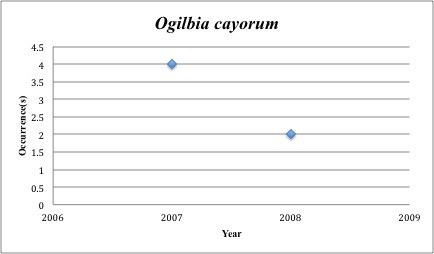
*Ogilbia cayorum*

**Figure 23.**
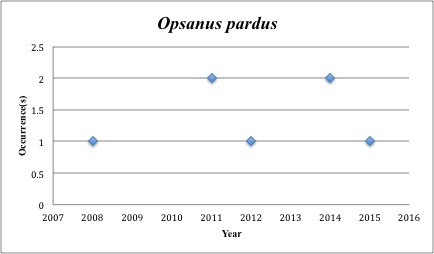
*Opsanus pardus*

**Figure 24.**
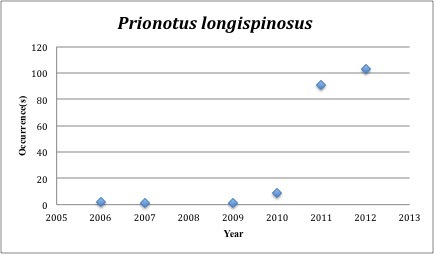
*Prionotus longispinosus*

**Figure 25.**
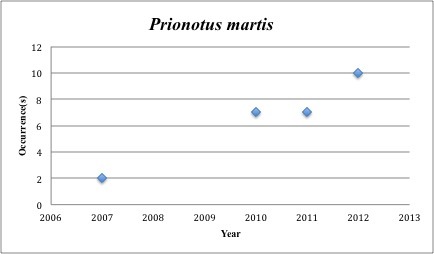
*Prionotus martis*

**Figure 26.**
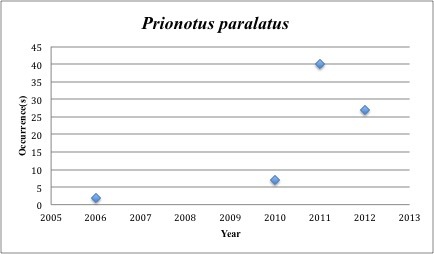
*Prionotus paralatus*

**Figure 27.**
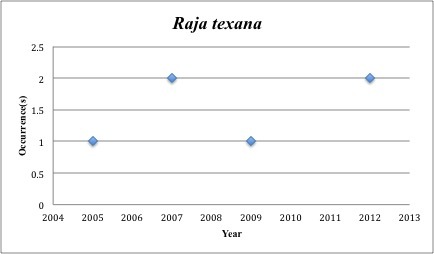
*Raja texana*

**Figure 28.**
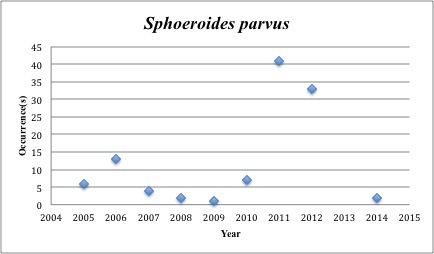
*Sphoeroides parvus*

**Figure 29.**
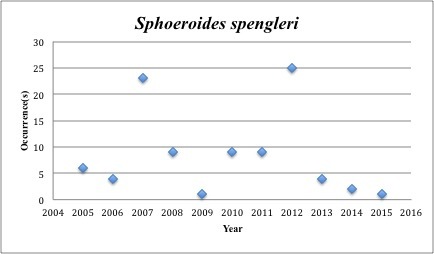
*Sphoeroides spengleri*

**Figure 30.**
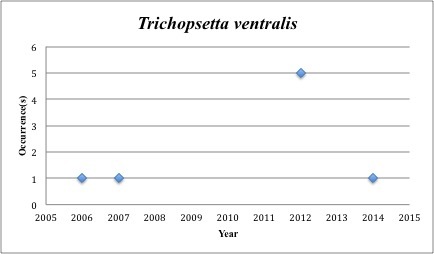
*Trichopsetta ventralis*

**Table 1.** Summary of species occurrence records (based on GBIF and FishNET2), and habitat types (from McEachran 2009; Chakrabarty et al. 2012). Taxa that were deemed ‘‘Species of Greatest Concern’’ by Chakrabarty et al. (2012) are in bold. These species had 35% of their historical occurrence records in the region of the oil spill.

**Species**: **Scientific name**	**Family**	**Occurrences: 2010-present**	**Occurrences**: **2005-present**	**Habitat**
*Alosa alabamae*	Clupeidae	12	24	Bay and Near Shore, Anadromous, Neritic
*Alosa chrysochloris*	Clupeidae	47	177	Bay and Near Shore, Anadromous, Neritic
***Anacanthobatis folirostris***	** Anacanthobatidae **	**0**	**0**	**Slope**
*Atherinella schultzi*	Atherinopsidae	1	1	Bay and Near Shore, Estuarine
*Atractosteus spatula*	Lepisosteidae	15	29	Bay and Near Shore, Neritic, Estuarine
***Bollmannia communis***	** Gobiidae **	**4**	**5**	**Demersal, Soft Substrates**
***Bollmannia eigenmanni***	** Gobiidae **	**0**	**0**	**Demersal**
*Brevoortia gunteri*	Clupeidae	9	17	Bay and Near Shore, Neritic, Estuarine
*Brevoortia patronus*	Clupeidae	85	180	Bay and Near Shore, Neritic, Estuarine
*Calamus arctifrons*	Sparidae	9	32	Demersal, Seagrass
*Calamus campechanus*	Sparidae	0	0	Demersal
*Chasmodes longimaxilla*	Blenniidae	0	0	Demersal, Coral Reef
	Gobiidae	0	0	Demersal
	Gobiidae	0	0	Demersal
*Citharichthys abbotti*	Paralichthyidae	0	0	Demersal, Soft Substrates
***Coryphaenoides mexicanus***	** Macrouridae **	**2**	**2**	**Benthopelagic, Slope, Abyssal**
*Coryphopterus punctipectophorus*	Gobiidae	0	0	Demersal, Coral Reef
*Ctenogobius claytonii*	Gobiidae	0	1	Demersal, Bay and Near Shore, Estuarine
*Cynoscion arenarius*	Sciaenidae	33	90	Demersal, Beach and Shoreline, Soft Substrates
*Dipturus olseni*	Rajidae	0	2	Demersal, Slope
	** Rajidae **	**0**	**0**	**Slope**
*Eptatretus minor*	Myxinidae	0	2	Slope, Soft Substrates, Burrower
***Eptatretus springeri***	** Myxinidae **	**1**	**1**	**Slope, Soft Substrates, Burrower**
	** Etmopteridae **	**0**	**5**	**Slope**
***Eustomias leptobolus***	** Stomiidae **	**0**	**0**	**Mesopelagic**
*Exechodontes daidaleus*	Zoarcidae	0	0	Benthic, Slope
*Floridichthys carpio*	Cyprinodontidae	3	17	Bay and Near Shore, Estuarine, Seagrass
*Fundulus grandis*	Fundulidae	97	292	Bay and Near Shore, Estuarine, Seagrass
*Fundulus jenkinsi*	Fundulidae	0	306	Bay and Near Shore, Estuarine
*Fundulus persimilis*	Fundulidae	0	2	Bay and Near Shore, Estuarine
*Fundulus pulvereus*	Fundulidae	35	69	Bay and Near Shore, Estuarine
*Fundulus xenicus*	Fundulidae	20	92	Bay and Near Shore, Estuarine
*Gambusia yucatana*	Poeciliidae	1	14	Bay and Near Shore, Estuarine
*Gobiosoma longipala*	Gobiidae	2	2	Demersal, Soft Substrates
*Gordiichthys ergodes*	Ophichthidae	0	3	Demersal, Burrower, Soft Substrates
*Gordiichthys leibyi*	Ophichthidae	0	0	Demersal, Soft Substrates, Burrower
***Gunterichthys longipenis***	** Bythitidae **	**0**	**0**	**Demersal, Bay and Near Shore, Burrower**
*Gymnachirus texae*	Achiridae	1	1	Demersal, Soft Substrates
*Halichoeres burekae*	Labridae	0	2	Coral Reef
***Halieutichthys intermedius***	** Ogcocephalidae **	**5**	**5**	**Benthic, Soft Substrates**
*Heteroconger luteolus*	Congridae	0	0	Demersal
***Hyperoglyphe bythites***	** Centrolophidae **	**0**	**1**	**Benthopelagic**
*Hypleurochilus caudovittatus*	Blenniidae	0	0	Demersal, Soft Substrates
*Hypleurochilus multifilis*	Blenniidae	0	0	Demersal, Coral Reef
*Ijimaia antillarum*	Ateleopodidae	0	0	Benthic, Slope
*Jordanella floridae*	Cyprinodontidae	19	40	Bay and Near Shore, Estuarine, Seagrass,
*Jordanella pulchra*	Cyprinodontidae	0	10	Bay and Near Shore, Estuarine
*Lepisosteus oculatus*	Lepisosteidae	84	146	Neritic, Bay and Near Shore, Estuarine
***Leucoraja lentiginosa***	** Rajidae **	**1**	**1**	**Demersal, Slope**
*Lupinoblennius nicholsi*	Blenniidae	0	0	Demersal
***Lycenchelys bullisi***	** Zoarcidae **	0	0	**Benthic, Slope**
*Menidia clarkhubbsi*	Atherinopsidae	0	0	Bay and Near Shore, Estuarine
*Menidia colei*	Atherinopsidae	0	29	Bay and Near Shore, Estuarine
*Menidia conchorum*	Atherinopsidae	0	0	Bay and Near Shore, Coral Reef
***Microdesmus lanceolatus***	** Microdesmidae **	0	0	**Demersal, Bay and Near Shore, Burrower**
*Monopenchelys acuta*	Muraenidae	0	5	Demersal, Coral Reef
***Mustelus sinusmexicanus***	** Triakidae **	**2**	**0**	**Soft Substrates**
*Neoopisthopterus cubanus*	Clupeidae	0	0	Neritic, Bay and Near Shore, Beach and Shoreline, Estuarine
*Ogcocephalus pantostictus*	Ogcocephalidae	6	6	Demersal
*Ogilbia cayorum*	Bythitidae	0	6	Demersal, Hard Substrate
***Oneirodes bradburyae***	** Oneirodidae **	0	0	**Bathypelagic**
*Ophichthus omorgmus*	Ophichthidae	0	0	Benthic, Slope, Soft Substrates
***Ophichthus rex***	** Ophichthidae **	**0**	**1**	**Demersal, Soft Substrates, Burrower**
***Opsanus pardus***	** Batrachoididae **	**6**	**7**	**Demersal, Hard Substrates**
***Parasaccogaster rhamphidognatha***	** Bythitidae **	0	0	**Benthic, Slope, Soft Substrates**
*Parmaturus campechiensis*	Scyliorhinidae	0	0	Slope, Soft Substrates
***Prionotus longispinosus***	** Triglidae **	**203**	**207**	**Demersal, Soft Substrates**
*Prionotus martis*	Triglidae	24	26	Demersal
*Prionotus paralatus*	Triglidae	74	76	Demersal, Benthic, Slope
*Raja texana*	Rajidae	2	6	Demersal
*Sanopus reticulatus*	Batrachoididae	0	0	Coastal Surface and Epipelagic, Demersal
*Sphoeroides parvus*	Tetraodontidae	83	109	Demersal, Bay and Near Shore
*Sphoeroides spengleri*	Tetraodontidae	50	93	Demersal, Coral Reef, Seagrass
*Stemonosudis bullisi*	Paralepididae	0	0	Mesopelagic
*Syngnathus affinis*	Syngnathidae	0	0	Benthopelagic, Bay and Near Shore, Seagrass
*Trichopsetta ventralis*	Bothidae	6	8	Demersal, Benthic, Soft Substrates
*Varicus marilynae*	Gobiidae	0	0	Demersal
